# Discovery of the small molecular inhibitors against TRPV6 as potential anti-cancer agents by structural based virtual screening

**DOI:** 10.3389/fchem.2026.1876502

**Published:** 2026-07-28

**Authors:** Lixuan Wang, Xin Deng, Ruoyang Hu, Shuguang Tan, Lin Zhang, Mingqiang Rong, Liqing Hu, Xing Feng, Hui Zou, Junbo Wu

**Affiliations:** 1 Hunan Laboratory of Study and Discovery of Small Targeted Molecules of Hunan Province, School of Pharmaceutical Sciences, Hunan Normal University, Changsha, China; 2 Engineering Research Center of Reproduction and Translational Medicine of Hunan Province, Hunan Normal University Health Science Center, Changsha, China; 3 Department of Colorectal Surgery, Affiliated Hengyang Hospital of Hunan Normal University & Hengyang Central Hospital, Hengyang, Hunan, China; 4 College of Life Sciences, Hunan Normal University, Changsha, China

**Keywords:** anti-cancer agents, inhibitors, structure-based virtual screening, TRPV6, tumor

## Abstract

TRPV6 is a member of the transient receptor potential cation channel family with selective permeability to Ca^2+^. Research indicates that TRPV6 is involved in the promotion of tumor cell proliferation and metastasis in various cancers, rendering it a prospective therapeutic target in cancer treatment. Thus, TRPV6 inhibitors are a very promising avenue for research into novel anti-tumor drugs. Nevertheless, the development of TRPV6 inhibitors remains in the preliminary stage, and the existing TRPV6 inhibitors demonstrate inadequate selectivity and off-target effects. Currently, the development of new drugs is being accelerated greatly by virtual screening technology, as is the reduction of research and development costs. In the present study, a novel TRPV6 inhibitor, designated AZ191, was identified through a structure-based virtual screening process utilizing the MCE and TOPSCIENCE databases. Electrophysiological experiments demonstrated that the compound AZ191 exhibited a high degree of affinity for hTRPV6, with an IC_50_ of 3.83 ± 0.32 μM. *In vitro* experiments have demonstrated the efficacy of compound AZ191 in combating tumors by virtue of its inhibitory effect on TRPV6. Notably, our study further demonstrates the good potential of TRPV6 inhibitors as novel anti-cancer drugs and provides a good lead compound for TRPV6 inhibitor research.

## 1 Introduction

To date, the Food and Drug Administration (FDA) and the National Medical Products Administration (NMPA) of China have approved 89 small-molecule targeted drugs for managing various oncological conditions (Bedard et al., 2020; Zho et al., 2021). However, there are some obvious shortcomings to current clinical antitumor drugs, such as easy drug resistance, off-target effects and limited efficacy. Researchers are constantly exploring new types of drugs to overcome these limitations. Examples include immunotherapy (e.g., PD-1 antibodies), antibody-drug conjugates (ADCs) and protein degradation technology (PROTACs), as well as synthetic lethality. Nonetheless, the primary focus of future cancer therapies will continue to be small-molecule targeted anticancer drugs. Continued innovation and development in this area will lead to more effective and personalized treatment strategies for cancer patients.

The transient receptor potential vanilloid 6 (TRPV6) is a highly selective calcium channel that plays a pivotal role in the following processes: extracellular calcium transport, calcium ion reuptake, and the maintenance of a local low-calcium environment (Gees et al., 2010; Lehen’Kyi et al., 2012; Hoenderop et al., 2003; den Dekker et al., 2003; Vennekens et al., 2000; Neuberger et al., 2025a). TRPV6 is predominantly expressed in non-excitable tissues, including the small intestine, epididymis epithelium, placenta, prostate, exocrine pancreas, kidney, and intestinal apical membranes (Nijenhuis et al., 2003a; Nijenhuis et al., 2003b; Zhuang et al., 2002). A body of extant literature has demonstrated a correlation between heightened TRPV6 expression and the incidence of diverse neoplasias (Bödding, 2007; Nilius et al., 2007; Prevarskaya et al., 2007; Santoni et al., 2011). The mechanism of action of TRPV6 in tumors is principally associated with the regulation of calcium-dependent proliferation, metastasis and apoptosis in cells (Lehen’kyi et al., 2007; Haustrate et al., 2024; Cordier et al., 2025). It is notable that TRPV6 expression is much higher in prostate, ovarian and pancreatic cancers than in normal tissue (Stewart, 2020). Prostate tumors that test positive for TRPV6 are more likely to spread and metastasize, which is often associated with a poor prognosis (Wissenbach et al., 2004). Increased expression of the TRPV6 protein has been found to correlate with a poor prognosis in patients with estrogen receptor-negative breast cancer (Peters et al., 2012). The development and prognosis of pancreatic cancer is also directly related to TRPV6, with patients who express high levels of the protein having a reduced chance of survival (Song et al., 2018). Therefore, TRPV6 plays an important role in the initiation and progression of cancer, making it a potential therapeutic target for a variety of cancers (Neuberger et al., 2023).

Several inhibitors targeting TRPV6 have recently been discovered, including macromolecular polypeptide, natural products and small-molecule compounds (Bowen et al., 2013; Fu et al., 2017; Xue et al., 2018). These inhibitors have demonstrated promising therapeutic potential in cancer treatment. Existing small-molecule inhibitors, such as 2-APB and TH-1177, have demonstrated promising channel activity and anti-tumor efficacy as allosteric inhibitors. However, these inhibitors only inhibit calcium transport at high micromolar concentrations and exhibit poor selectivity (Xu et al., 2016). Research into direct-blocking inhibitors, such as PCHPDs and Ru complexes, is still in its early stages because of the instability and possible negative side effects of these inhibitors (Cunha et al., 2020; Simonin et al., 2015). These findings highlight the need for further in-depth research into small-molecule inhibitors in order to unlock their potential for cancer treatment. Meanwhile, it is of great importance to develop efficient and highly selective TRPV6 inhibitors.

Virtual screening technology offers the advantages of high throughput, efficiency and low cost. This can significantly reduce the time and expense involved in drug research and development. In 2017, the crystal structure of the human TRPV6 (PDB: 6BO8) was first revealed (Saotome et al., 2016). Further research has revealed that the amino acid residues Ile575, Asp580 and Trp583 are the key sites responsible for regulating the function of TRPV6 (Bhardwaj et al., 2020). Therefore, based on the above research, this article used virtual screening techniques to search for novel TRPV6 inhibitors with anti-tumor activity.

In this work, approximately 250,000 small molecular compounds from the MCE and TOPSCIENCE databases were firstly screened with the TRPV6 channel. Those that formed stable interactions with the Ile575, Asp580 orTrp583 residues of the TRPV6 protein were then filtered out. Secondly, we selected the top 50 compounds based on the scoring values. Given the structural diversity of the compounds and the binding affinity for TRPV6, six compounds were manually selected and purchased from the original set of 50. Subsequently, we then evaluated their inhibitory activity against TRPV6 using electrophysiological experiments. Finally, we successfully identified the novel TRPV6 inhibitor AZ191, which has an IC_50_ of 3.83 ± 0.32 μM and demonstrates good selectivity. *In vitro* experiments have demonstrated the efficacy of AZ191 in combating tumors due to its inhibitory effect on TRPV6. In summary, the present study provides further evidence for the potential of TRPV6 inhibitors as anti-cancer agents. In addition, it identifies a novel and promising lead compound, AZ191, for research into TRPV6 inhibitors.

## 2 Results and discussion

### 2.1 Virtual screening based on the structure of human TRPV6

**FIGURE 1:**
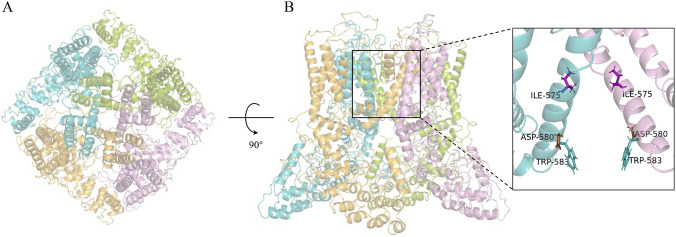
Structural diagram of the human TRPV6 channel protein (PDB ID: 6BO8). **(A)** Cross‐section of human TRPV6 channel protein crystal structure. **(B)** Crystal structure of the human TRPV6 channel protein with a vertical cross‐section, as well as an enlarged view of the binding site for the ligand screening. ILE‐575: Isoleucine‐575, ASP580: Aspartic Acid‐580, TRP‐583: Tryptophan‐583.

The human TRPV6 protein was obtained from the Protein Data Bank (PDB) (PDB ID: 6BO8). This channel protein consists of four subunits (Figure 1A), each of which consists of 725 amino acids. The focus of virtual screening is on TRPV6 channels with the key residues Ile575, Asp580 and Trp583 (Figure 1B), and the receptor proteins must undergo water pretreatment and hydrogenation. The anti-cancer activity databases employed in this virtual screening process contain 250,000 compounds, which are sourced from two distinct sources, namely,: MCE and TOPSCIENCE. The preliminary processing of small molecules was conducted utilizing the Schrodinger software, thereby yielding a self-constructed database comprising a range of small molecule conformations. Moreover, the virtual screening process ensures that the target compounds will interact with the key residues Ile575, Asp580 or Trp583 through the formation of hydrogen bonds or other interaction forces. The docking scoring results demonstrated that the top 50 small molecules exhibited a high degree of affinity for the TRPV6 channel molecules during the docking process. Consequently, the top 50 compounds (Table 1) with docking scores were selected for subsequent manual screening.

**TABLE 1:** Lists the molecular docking results of the top 50 compounds.

Ranking	Name	Scores
1	Adrenorphin	−13.50
2	Polymyxin B nonapeptide (TFA)	−13.26
3	SPR741 (acetate)	−12.77
4	Birinapant	−12.64
5	MS4078	−12.64
6	Dermorphin	−12.56
7	TRV120055	−12.51
8	UNC0646	−12.51
9	Mogroside IV-A	−12.44
10	BSJ-4-116	−12.43
11	Isavuconazonium (sulfate)	−12.33
12	Hexa-His	−12.32
13	Gamitrinib TPP hexafluorophosphate	−12.28
14	Epimedin B	−12.14
15	Protease-activated Receptor-4	−12.10
16	PCI-33380	−12.08
17	BSJ-4-116	−12.06
18	Transdermal peptide disulfide (TFA)	−12.04
19	Batefenterol	−11.88
20	[Sar1, Ile8]-Angiotensin II (TFA)	−11.86
21	ACBI1	−11.82
22	AZD4320	−11.78
23	Tilmicosin	−11.72
24	ABT-737	−11.70
25	Pan-RAS-IN-1	−11.69
26	KU 59403	−11.62
27	PROTAC CDK2/9 Degrader-1	−11.59
28	Revefenacin	−11.59
29	Rilapladib	−11.58
30	Tubuloside A	−11.54
31	SRX3207	−11.53
32	UNC 0631	−11.52
33	Tildipirosin	−11.52
34	BAY-850	−11.50
35	WRW4	−11.48
36	PROTAC CDK2/9 Degrader-1	−11.46
37	Caspofungin (acetate)	−11.45
38	Deltarasin	−11.44
39	AZ191	−11.26
40	DAPTA	−11.24
41	Olcegepant	−11.03
42	Isoliensinine	−11.01
43	URMC-099	−11.01
44	TRV120055	−10.99
45	Saquinavir	−10.94
46	Pinometostat	−10.89
47	GSK1904529A	−10.80
48	TCS7010	−10.76
49	Isoquercetin	−10.49
50	DDR1-IN-1	−10.48

### 2.2 Manual re-screening based on virtual screening results

**FIGURE 2:**
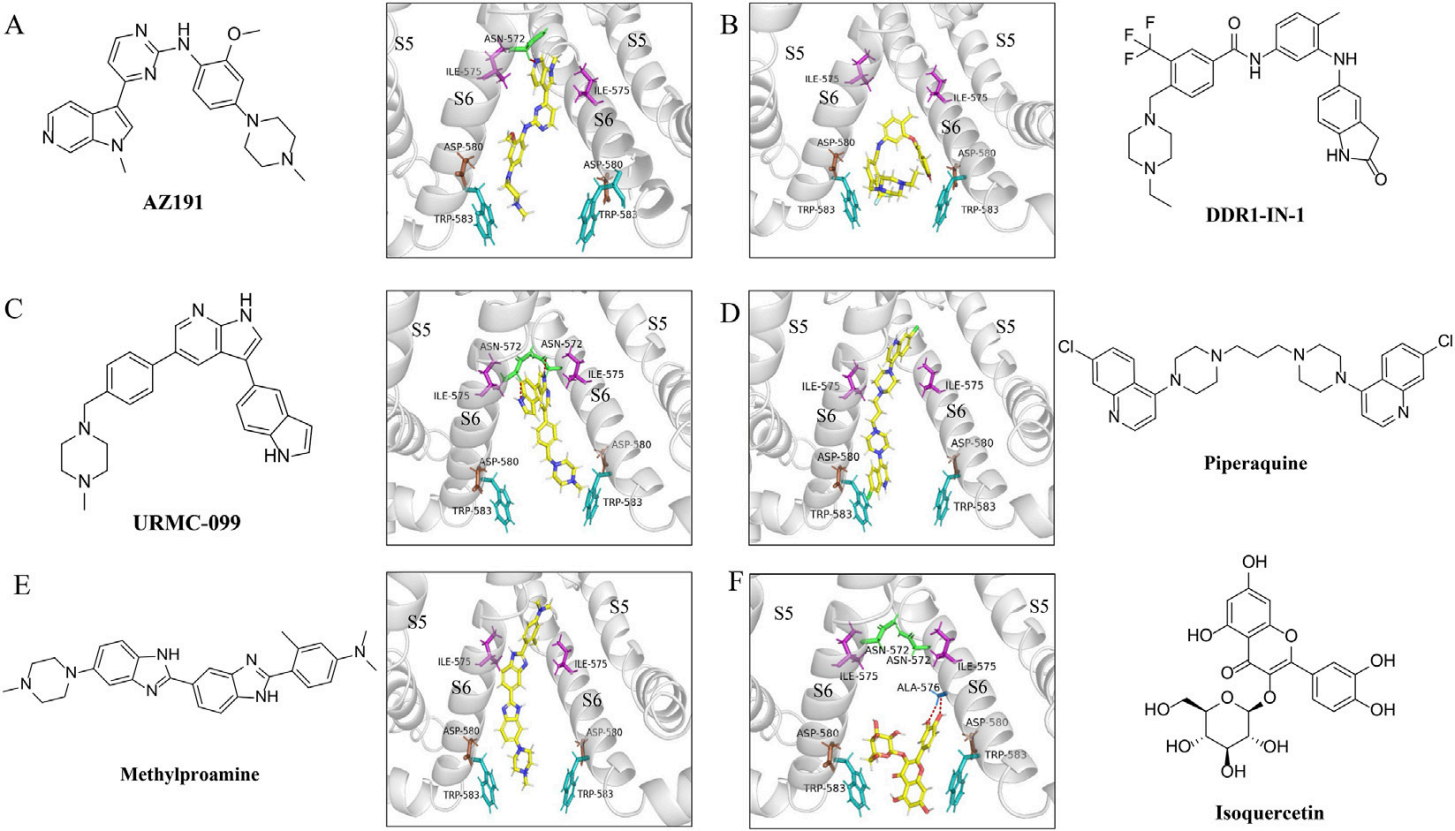
The TRPV6 protein (PDB ID: 6BO8) is illustrated in white cartoon form, the ligand small molecule is shown in yellow rod structure, the hydrogen bond is shown in red dashed line, and the key amino acid residues are shown in rod structure. **(A)** The structure of the TRPV6/AZ191 complex. **(B)** The structure of the TRPV6/DDR1-IN-1 complex. **(C)** The structure of the TRPV6/URMC-099 complex. **(D)** The structure of the TRPV6/methylproamine complex. **(E)** The structure of the TRPV6/piperaquine complex. **(F)** The structure of the TRPV6/isoquercetin complex.

During structure-based virtual screening, the TRPV6 protein was treated as a rigid structure. Conversely, small molecules in the compound library generate numerous compound structures with different conformations during ligand preparation. During docking, the binding ability of small molecules with different conformations to channel proteins varies. These molecules are ranked by docking score, from high to low. The docking score is related to the binding free energy. Moreover, the structures of the top 50 compounds were found to be simple, and the combination of model compounds was deemed to be unreasonable. Consequently, further analysis of the docking results is required using artificial selection. In accordance with the aforementioned screening methodologies, a selection of six small-molecule compounds (Figure 2) was identified from the top 50, with the subsequent experimental verification of their docking scores.

It is evident from the observation of the structure of the six small molecules illustrated in Figure 2 that they are all heterocyclic compounds, characterized by aromatic properties. Furthermore, these compounds exhibit structural similarity to the small molecule inhibitors of the TRPV6 channel, including 2-APB and cis-22a (Hofer et al., 2013; Singh et al., 2018). As demonstrated in Figure 2, it was established that all of these structures (AZ191, DDR1-IN-1, URMC-099, Methylproamie, Piperaquine and Isoquercitrin) exhibited good steric complementarity with the TRPV6 binding site, forming hydrogen bonds or π-π interactions with key amino acid residues. Actually, AZ191 has been reported to be a potent DYRK1B inhibitor with significant anti-tumor activity. The results of the present study suggest the potential for these compounds to act as inhibitors of TRPV6. However, further research is required to verify this hypothesis.

### 2.3 Study of the TRPV6 activity of candidate compounds

**FIGURE 3:**
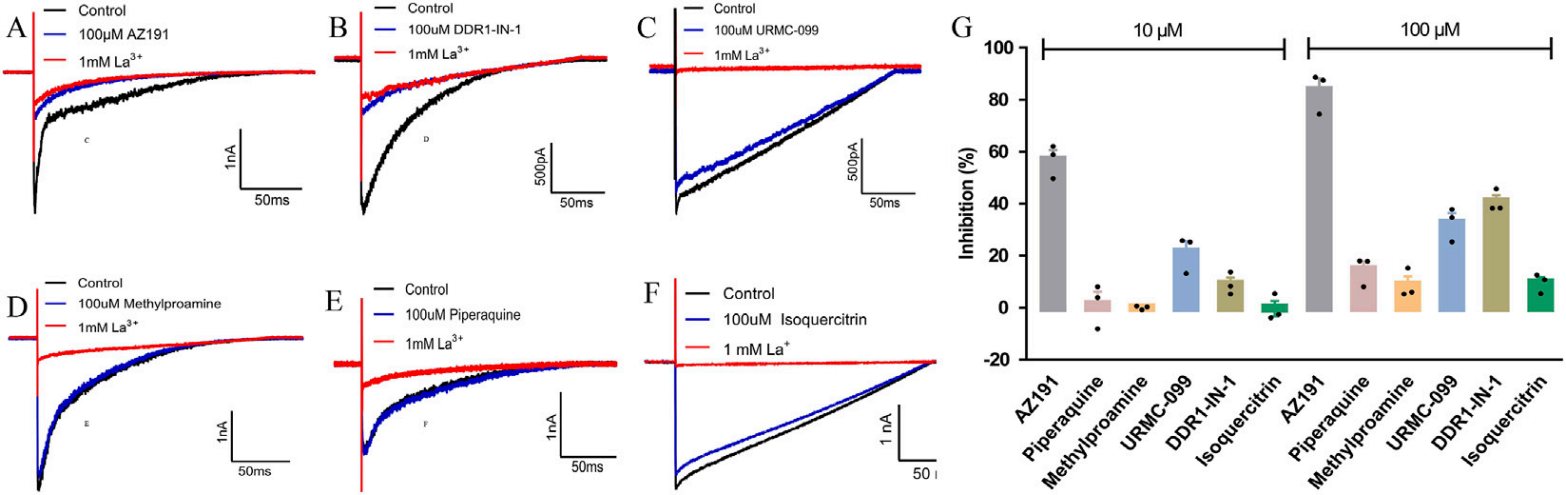
Analysis of TRPV6 activity of candidate compounds. **(A–F)** Representative current plots of human TRPV6 before and after treatment with 100 µM of the candidate compounds (black: current before treatment; blue: current of TRPV6 channel treated with 100 µM of the candidate compounds; red: current from 1 mM of La^3+^ acting on TRPV6 channel). **(G)** The bar graph shows the inhibition of TRPV6 channel current by 100 µM of the candidate compounds. Data are presented as the mean ± standard error, *n* = 3.

In order to preliminarily evaluate the potential of the compounds obtained from the aforementioned screening to inhibit TRPV6 activity, an electrophysiological experiment was conducted. We first purchased the six compounds from the TOPSCIENCE company. The extracellular solution was prepared using dimethyl sulfoxide (DMSO) as the solvent for the 100 mM stock solution. This was then diluted to create working solutions at concentrations of 100 µM and 10 μM, respectively, for activity testing. Then, electrophysiological experimentation was conducted in accordance with the following parameters: a muzzle voltage of 0 mV was maintained, with the application of 100 mV intervals of 5 s and +100 mV slopes, with each slope stimulus completed within 200 ms. The results showed that the six small molecules could obviously inhibit the TRPV6 channel at a concentration of 100 μM. Specifically, as demonstrated in Figure 3, AZ191 exhibited the most significant inhibitory effect on TRPV6 channel currents, with an average inhibition of 74.55% ± 9.19% at a concentration of 100 μM. This was followed by DDR1-IN-1 (38.28% ± 7.76%) and URMC-099 (20.26% ± 10.77%). The study found that piperaquine, methylproamine and isoquercitrin exhibited suboptimal inhibition of the channel current, with values of 8.09% ± 9.02%, 5.33% ± 3.68% and 10.84% ± 3.46%, respectively. The above findings preliminarily confirm that small-molecule ligands with a V-shaped spatial structure exhibit superior inhibitory activity against TRPV6, as seen in compounds such as AZ191, URMC-099, and DDR1-IN-1. Thus, we selected compound AZ191 for further experimentation in order to assess its activity and selectivity towards TRPV6.

### 2.4 The ADMET properties of AZ191

To preliminarily assess the potential of compound AZ191 as a lead compound, its ADMET properties were predicted. Table 2 presents the predicted ADMET properties of AZ191, showing good intestinal absorption (99.7%) and high Caco-2 permeability. The compound is both a P-glycoprotein substrate and inhibitor (types I and II), indicating potential for efflux and drug-drug interactions. It exhibits moderate water solubility and low skin permeability. Regarding distribution, AZ191 has a high volume of distribution (VDss 1.411 log L/kg) and an unbound fraction of 0.21, but shows very low permeability across the blood-brain barrier (BBB) and the CNS. It is a CYP3A4 substrate and inhibitor, but does not impact several other CYP enzymes. The total clearance is moderate, and it is not a renal OCT2 substrate. However, significant toxicity risks are noted: AZ191 is predicted to be Ames-positive (mutagenic), hepatotoxic, and a hERG II inhibitor (cardiac risk). It also has a relatively low predicted maximum tolerated dose in humans, while acute oral toxicity in rats is moderate. Skin sensitization is not expected, and aquatic toxicity is low to moderate.

**TABLE 2:** ADMET properties of AZ191.

Properties	Model name	Predicted values	Units
Absorption	Water solubility	−3.196	Numeric (log mol/L)
	Caco2 permeability	1.228	Numeric (log papp in 10–6 cm/s)
	Intestinal absorption (human)	99.707	Numeric (% absorbed)
	Skin permeability	−2.753	Numeric (log Kp)
	P-glycoprotein substrate	Yes	Categorical (yes/No)
	P-glycoprotein I inhibitor	Yes	Categorical (yes/No)
	P-glycoprotein II inhibitor	Yes	Categorical (yes/No)
Distribution	VDss (human)	1.411	Numeric (log L/kg)
	Fraction unbound (human)	0.21	Numeric (Fu)
	BBB permeability	−0.894	Numeric (log BB)
	CNS permeability	−2.9	Numeric (log PS)
Metabolism	CYP2D6 substrate	No	Categorical (yes/No)
	CYP3A4 substrate	Yes	Categorical (yes/No)
	CYP1A2 inhibitor	No	Categorical (yes/No)
	CYP2C19 inhibitor	No	Categorical (yes/No)
	CYP2C9 inhibitor	No	Categorical (yes/No)
	CYP2D6 inhibitor	No	Categorical (yes/No)
	CYP3A4 inhibitor	Yes	Categorical (yes/No)
Excretion	Total clearance	0.677	Numeric (log mL/min/kg)
Excretion	Renal OCT2 substrate	No	Categorical (yes/No)
Toxicity	AMES toxicity	Yes	Categorical (yes/No)
Toxicity	Max. Tolerated dose (human)	−0.236	Numeric (log mg/kg/day)
Toxicity	hERG I inhibitor	No	Categorical (yes/No)
Toxicity	hERG II inhibitor	Yes	Categorical (yes/No)
Toxicity	Oral Rat acute toxicity (LD50)	3.011	Numeric (mol/kg)
Toxicity	Oral Rat chronic toxicity (LOAEL)	1.104	Numeric (log mg/kg_bw/day)
Toxicity	Hepatotoxicity	Yes	Categorical (yes/No)
Toxicity	Skin sensitization	No	Categorical (yes/No)
Toxicity	Pyriformis toxicity	0.286	Numeric (log ug/L)
Toxicity	Minnow toxicity	3.021	Numeric (log mM)

AZ191 exhibits excellent intestinal absorption, high permeability, and wide tissue distribution (high VDss), but it struggles to cross the blood–brain barrier. Its safety concerns are significant: a positive AMES test indicating mutagenicity, hERG II inhibition suggesting cardiotoxicity, hepatotoxicity, and a low predicted maximum tolerated dose. Its profile as a P-gp substrate/inhibitor, and its interaction with CYP3A4, also suggest possible drug–drug interactions. Due to these toxicity issues, AZ191 should be used at very low doses to reduce off-target effects or formulated as targeted nanoparticles to deliver it specifically to diseased cells, avoiding healthy tissues. These approaches are essential to mitigate its genotoxic, cardiotoxic, and hepatotoxic risks, which could otherwise impede its development as a therapeutic agent. Hence, these data indicate that compound AZ191 has the potential to serve as a lead compound for further research.

### 2.5 The activity and selectivity of the TRPV6 channel for compound AZ191

**FIGURE 4:**
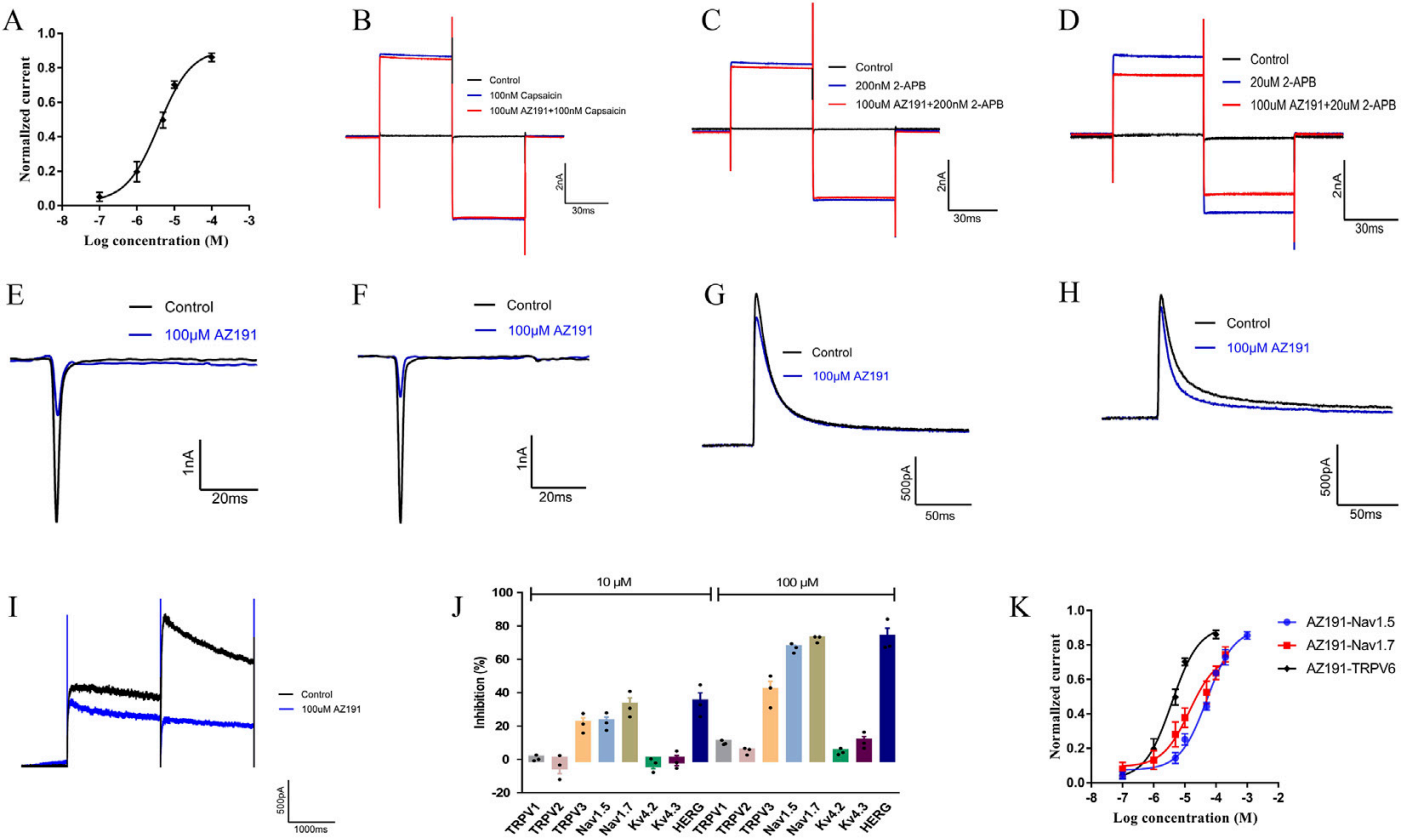
Activity and selectivity analysis of compound AZ191 on TRPV6 channel. **(A)** The concentration effect curve of AZ191 on TRPV6 channel. **(B–I)** The representative current plots of TRPV1, TRPV2, TRPV3, Na_V_1.5, Na_V_1.7, K_V_4.2, K_V_4.3, and HERG channels inhibited after treatment with 100 μM AZ191. **(J)** The statistical plots demonstrate the inhibition of the above-mentioned channels by AZ191 at concentrations of 10 and 100 µM. **(K)** The concentration effect curves of AZ191 on NaV1.5 and NaV1.7 channels. Data are presented as mean ± standard error, *n* = 6-8.

Further studies targeting the TRPV6 channel with AZ191 have shown that it has a strong affinity for the channel, with an IC_50_ value of 3.83 ± 0.32 μM (Figure 4A). Indeed, NaV1.5, KV4.2 and KV4.3 are expressed in abundance in the heart and brain. These ions are indispensable for the formation of cardiac action potentials and the regulation of neuronal excitability. Furthermore, it has been demonstrated that dysfunction of the potassium voltage-gated channel subfamily 4 (KV4) can result in the onset of behavioural disorders, epilepsy, myocardial infarction and other diseases (Long et al., 2005; Smith et al., 2016). Consequently, developing small-molecule drugs that selectively target the TRPV6 channel poses a challenge in terms of channel subtype selectivity. To avoid toxicity, it is important to minimize the presence or reduction of inhibitory activity of the Na_V_1.5, K_V_4.2 and K_V_4.3 channels.

Accordingly, an additional investigation was therefore conducted to examine the selectivity of the compound AZ191 with a focus on TRPV6. As shown in Figures 4B–J. The findings indicate that at a concentration of 100 μM, AZ191 demonstrated weak sensitivity to TRPV1, TRPV2, TRPV3, K_V_4.2, and K_V_4.3 channels, with inhibition rates recorded as 9.42% ± 3.31%, 6.59% ± 2.47%, 42.90% ± 10.84%, 4.20% ± 2.74%, and 9.70% ± 5.69%, respectively. The inhibition rates of Na_V_1.5, Na_V_1.7 and HERG were found to be 67.16% ± 3.64%, 73.49% ± 2.54% and 70.85% ± 10.31%, respectively. Furthermore, the results demonstrated the concentration-effect curves of AZ191 for the Na_V_1.5 and Na_V_1.7 channel subtypes at a holding voltage of −90 mV. The IC_50_ of AZ191 was found to be 51.38 ± 0.32 μM for the Na_V_1.5 channel and 14.13 ± 0.51 μM for the Na_V_1.7 channel (Figure 4K). Hence, the results outlined above indicate that compound AZ191 demonstrates a suboptimal level of selectivity for TRPV6 and carries an inherent risk of side effects and hepatotoxicity, it can nevertheless serve as a lead compound for the development of TRPV6 inhibitors. This facilitates the subsequent optimization of structure to enhance selectivity and mitigate the potential adverse effects.

### 2.6 Analysis of AZ191 activity on TRPV6 channel chimeras and mutants

**FIGURE 5:**
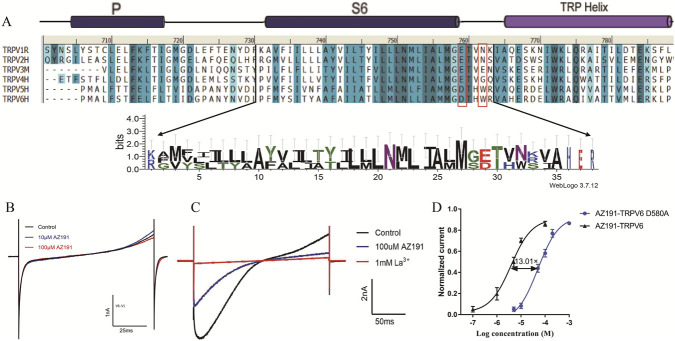
**(A)** Sequence alignment of the S6 fragment of the TRPV channel and its flanking proteins. **(B)** The current map of the TRPV6/TRPV1 chimera in the presence of AZ191. **(C)** The figure illustrates the capacity of AZ191 to impede the activity of the TRPV6 D580A mutant channel. **(D)** The concentration-dependent effects of AZ191 on TRPV6 channels and the TRPV6 D580A mutant channel. Data are presented as mean ± standard error, *n* = six to eight.

Previous results showed that AZ191 was insensitive to TRPV1 and TRPV2 channels at a test concentration of 100 μM, with inhibition rates of 9.42% ± 3.31% and 6.59% ± 2.47%, respectively. A sequence alignment analysis was performed on proteins associated with the pore region of TRPV1, TRPV2, TRPV3, TRPV4, TRPV5 and TRPV6. The analysis revealed a certain degree of similarity in the amino acid sequence. However, a notable variation was observed in the amino acids 580 and 583 among TRPV1, TRPV2 and TRPV6, indicating a potential divergence in their functional characteristics (Figure 5A). Of these, TRPV1 and TRPV2 both had a glutamate residue at position 580 and an asparagine residue at position 583. In contrast, TRPV6 had an aspartate residue at position 580 and a tryptophan residue at position 583 (Figure 5A). Both glutamate and aspartate are classified as basic amino acids. Meanwhile, asparagine and tryptophan are considered polar amino acids. Although the amino acid properties of the two key sites are similar in the different isoforms, differences in their side-chain structure may affect the conformation of the channel, resulting in variations in their affinity for AZ191.

To further explore the interaction mechanism between the compound AZ191 and the pore region of the TRPV6 channel, particularly the S6 segment (Figure 5A), TRPV6/TRPV1 and TRPV6/TRPV2 S6 channel chimeras were constructed. The electrophysiological results showed that AZ191 inhibited the S6 channel mosaic current of the TRPV6/TRPV1 by 5.38% ± 3.71% at a concentration of 100 μM, which is much lower than AZ191’s affinity for wild-type TRPV6 channel under equivalent conditions (Figure 5B). Additionally, no significant current was detected by TRPV6/TRPV2 S6 channel chimeras. It is reasonable to speculate that the S6 fragment of the TRPV6 channel is important when AZ191 binds to TRPV6.

To further understand the mechanism of action of AZ191 on TRPV6 channel, we explored its effects on the activity of mutant TRPV6 channels. The construction of three-channel mutants (I575A, D580A and W583A) was informed by the findings of the chimera experiment and the key amino acids that were used in virtual screening (Liu et al., 2024; Neuberger et al., 2025b). The results of electrophysiological experiments demonstrated that the current characteristics of the channel mutants remained unaltered when the key amino acid D580 was mutated. However, the compound AZ191 was observed to induce a decrease in the activity of the channel mutants (Figure 5C). Especially, the IC_50_ of AZ191 on the human TRPV6 D580A mutant channel was determined to be 49.91 ± 0.02 μM (Figure 5D). Furthermore, AZ191 was found to exhibit a 13.01-fold decrease in activity for the D580A mutant TRPV6.

However, when the W583A mutant was introduced into normal HEK-293T cells, their morphology changed significantly and they grew into exfoliated spherical cells. This is consistent with the results obtained by Dang et al. in their TRPV5 experiments (Dang et al., 2019; Hughes et al., 2018; van der Wijst et al., 2017). The occurrence of the W583A mutation results in the continuation of channel development and an increase in calcium influx, leading to cell death. It is reasonable to hypothesize that the occurrence of a W583A mutation in the TRPV6 will result in the sustained opening of the channel, which may ultimately lead to cell death. Moreover, the TRPV6 I575A mutant plasmid exhibited an inability to record currents following transfection of cells. The results presented herein indicate that the activity of AZ191 on TRPV6 was significantly reduced when aspartate residue at position 580 was mutated to alanine. This indicates that this site is crucial for the function of AZ191, and further verifies the rationale behind selecting it for compound screening. Conversely, subsequent structural optimization using AZ191 as a lead compound will facilitate simultaneous consideration of interactions with the W583 and I575 sites. This may result in a substantial enhancement of inhibitory activity and selectivity against TRPV6.

### 2.7 TRPV6 is instrumental in the genesis of a plethora of cancers

**FIGURE 6:**
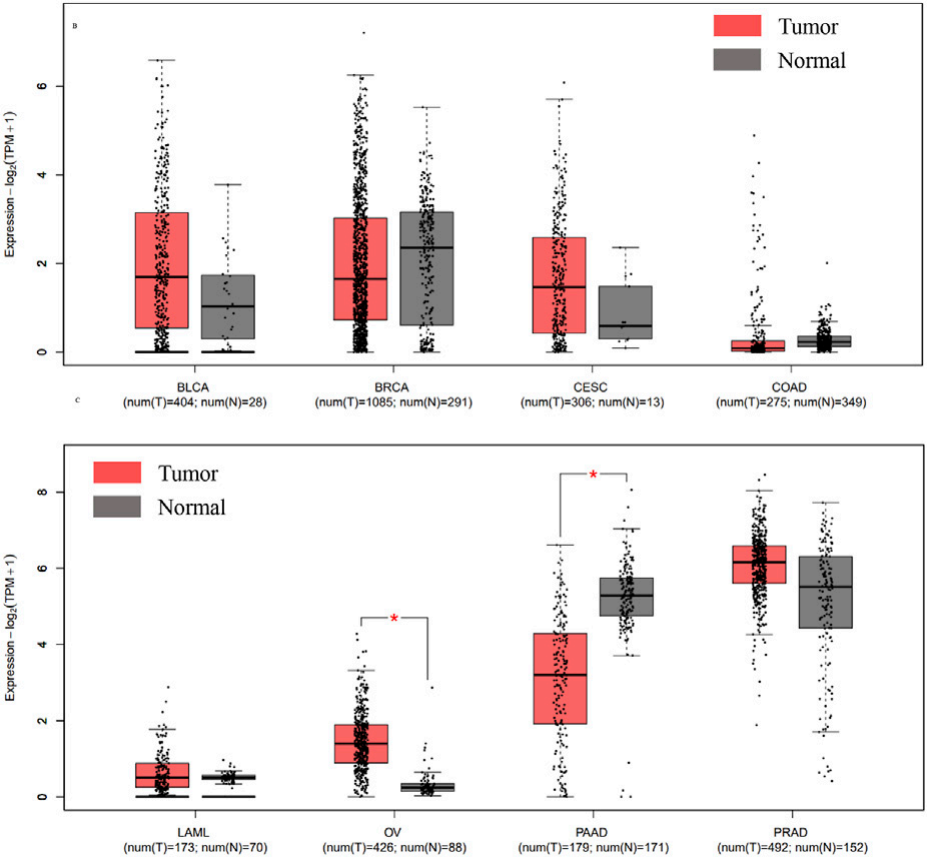
Expression of the TRPV6 gene in bladder urothelial carcinoma, breast cancer, cervical cancer, colorectal cancer, myeloid leukaemia, ovarian cancer, pancreatic cancer and prostate cancer cells, as well as in their corresponding normal cells. The data presented herein originates from the TCGA and GTEx databases. The gene expression profile map was produced by the interactive gene expression profile analysis tool GEPIA.

To further investigate the role of TRPV6 in tumor development, we examined its expression levels in various tumors using bioinformatics. Specifically, GEPIA (a gene expression analysis software) was used to analyze TRPV6 gene expression obtained from the TCGA and GTEx databases in various cancerous and normal tissues, in order to determine the differences in TRPV6 gene expression between tumor cells and normal tissues. The analysis results demonstrated that the TRPV6 gene exhibited elevated and atypical levels of expression in various types of cancer, including bladder urothelial cancer (BLCA), breast cancer (BRCA), cervical cancer (CECS), colon adenocarcinoma (COAD), myeloid leukaemia (LAML), ovarian cancer (OV), and prostate cancer (PRAD). However, in the case of pancreatic cancer (PAAD), the expression levels of the TRPV6 gene were found to be comparable to those observed in normal tissues. (Figure 6). The present study has demonstrated that the expression level of the TRPV6 gene is significantly increased in tumor cells. This finding suggests a potential correlation between the expression of the TRPV6 gene and the occurrence and development of tumors. Thus, these data strongly support the malignant role of TRPV6 in tumours and provide robust evidence for selecting cell lines for the subsequent evaluation of AZ191’s anti-tumor activity.

### 2.8 Compound AZ191 displays broad-spectrum anti-tumor activity

**FIGURE 7:**
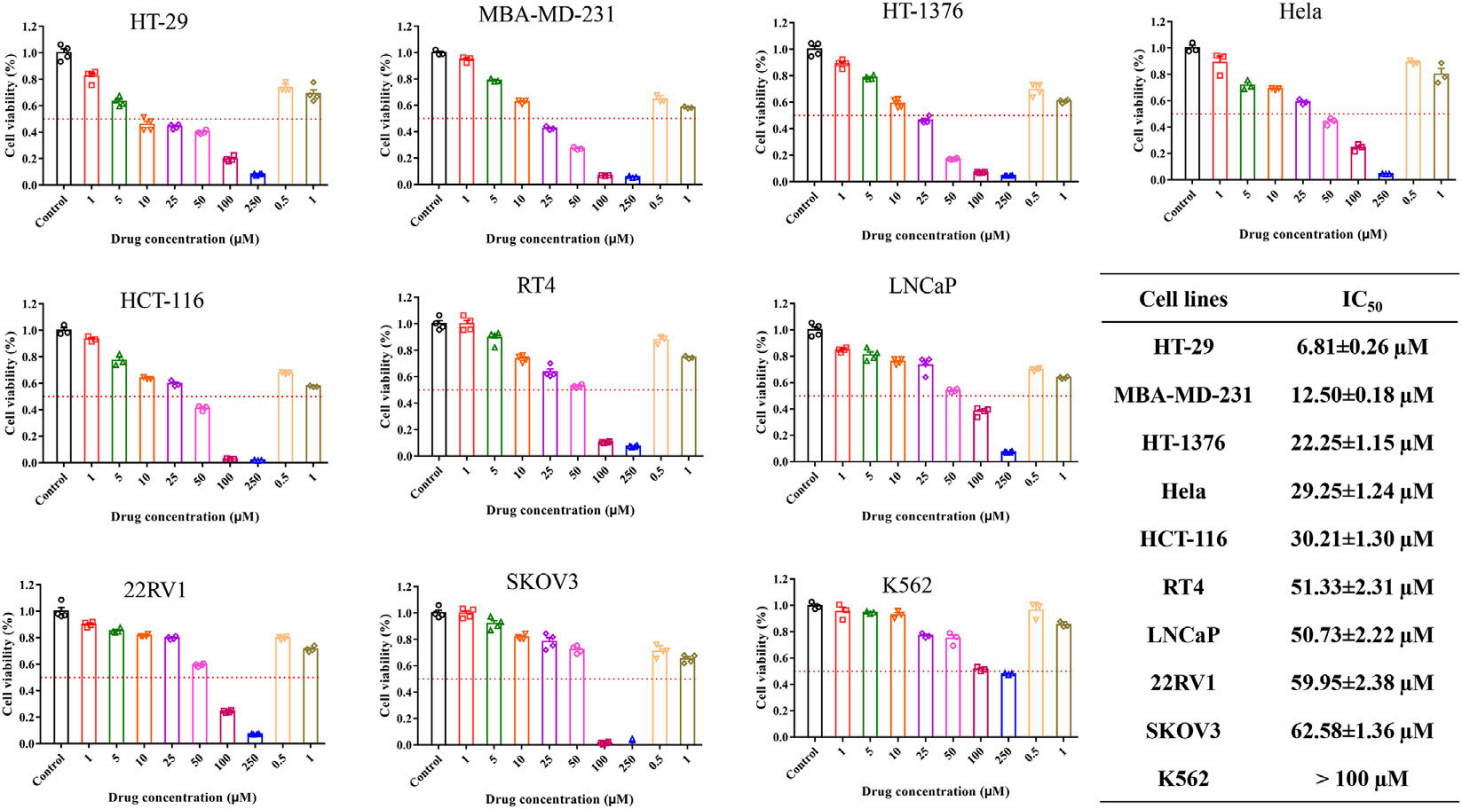
Compound AZ191 demonstrated a significant inhibitory effect on the proliferation of tumor cells, exhibiting a concentration-dependent response. The concentrations of paclitaxel, which were set at 0.5 and 1 μM, are shown on the right of each figure. Data are expressed as mean ± standard error (n = 3 or 4 independent experiments).

In view of the important oncogenic role of TRPV6 in numerous tumors and the potent inhibitory activity of AZ191 against TRPV6, the anti-tumor activity of the compound AZ191 was subsequently evaluated. Following comprehensive analysis of bioinformatics data, the following cell lines were selected for further investigation: bladder cancer cells (RT4, HT-1376), triple-negative breast cancer cells (MBA-MD-231), colorectal cancer cells (HCT-116, HT-29), cervical cancer cells (Hela), ovarian cancer cells (SLOV3), prostate cancer cells (LNCaP, 22RV1) and chronic myeloid leukaemia cells (K562). The proliferation-inhibitory effect of AZ191 was subjected to a systematic evaluation, with the above cell lines utilized for the purpose. The concentration of compound AZ191 was established at a range of 1–250 μM, while the concentration of the positive control, paclitaxel, was set at 0.5 and 1 µM (Figure 7). The experimental findings demonstrated that the compound AZ191 exhibited a substantial inhibitory effect on the proliferation of tumor cells, exhibiting a concentration-dependent response (Figure 7). Compound AZ191 demonstrated the greatest activity in inhibiting the proliferation of HT-29 cells, with an IC_50_ of 6.81 ± 0.26 µM. This was followed by MDA-MB-231 cells, with an IC_50_ of 12.50 ± 0.18 µM AZ191 exhibited comparable inhibitory activity against the HT-1376, Hela and HCT-116, cell lines, with respective IC_50_ values of 22.25 ± 1.15 µM, 29.25 ± 1.24 µM and 30.21 ± 1.30 µM, respectively. By contrast, the inhibitory activity of compound AZ191 was relatively weaker against the RT4, LNCaP, 22RV1 and SKOV3 cell lines, with respective IC_50_ values of 51.33 ± 2.31 µM, 50.73 ± 2.22 µM, 59.95 ± 2.38 µM and 62.58 ± 1.36 µM. Bioinformatics analysis revealed that TRPV6 was overexpressed in the aforementioned tumors. The TRPV6 inhibitor AZ191 was found to effectively inhibit the proliferation of these cells, thus demonstrating the significant anti-tumor effect of TRPV6 inhibition. It is noteworthy that the experimental findings demonstrate that compound AZ191 displays optimal inhibitory activity against the microsatellite-stable colorectal cancer cell line HT-29, while its inhibitory effect on the microsatellite-unstable cell line HCT-116 is comparatively low. This finding suggests the potential for disparities in TRPV6 expression levels or function between these 2 cell lines, which necessitates further targeted research. The study also revealed that AZ191 had little inhibitory effect on K562 cells, with an IC_50_ value of over 100 µM (Figure 7). This phenomenon may be attributable to the hypothesis that TRPV6 exerts a more substantial role in solid tumors than in hematological malignancies. This supposition requires further elucidation through subsequent in-depth studies. In order to further investigate whether the V-shaped structure of the AZ191 compound is significantly correlated with its ADMET properties and anti-tumor activity, we are currently conducting structural optimization work using AZ191 as a lead compound. In conclusion, the preceding studies further illustrate that AZ191 exerts a broad-spectrum antitumor effect by inhibiting TRPV6.

## 3 Experiment

### 3.1 Virtual screening

The crystal structure of TRPV6 channels was obtained from the online protein database, RCSB PDB (https://www.rcsb.org/) with PDB ID 6BO8. In this study, the Glide module of Schrodinger software was used to perform molecular docking. Unlike other docking programs, which dock ligands to the rigid 3D structures of known protein receptors, Glide uses a series of hierarchical filters to systematically search the conformations, orientations and positions of docking ligands. Search for its possible location in the active site region of the receptor. According to the different shapes and features of the field set, the receptor is treated in the pre-treatment of the computing steps to generate it, so that it is calculated only once for each receptor. The initial rough localization and scoring stage involves an exhaustive enumeration of the minimum values in ligand torsional angle space, which can significantly narrow the search space. Then, for a certain number of surviving conformations, the standard molecular mechanics energy function, combined with the distant-dependent dielectric model, is used to optimize the torsional flexibility of the ligand within the receptor field. Finally, the best candidate is further refined through Monte Carlo sampling of the conformations. In some cases, this is crucial to obtain accurate docking postures, and the optimal docking posture is selected using a combination of empirical and force field-based model energy functions (Friesner et al., 2004).

There are three Glide docking methods: High Throughput Virtual Screening (HTVS), Standard-Precision (SP) and Extra-Precision (XP). Glide HTVS docks compounds at a rate of about 2 seconds per compound, while Glide SP performs exhaustive sampling, balancing speed and precision at about 10 seconds per compound. Glide XP uses anchoring and growth sampling methods, taking different functional forms for GlideScore. It takes about 2 min per compound to dock them (https://www.schrodinger.com/science-articles/docking-and-scoring). Considering the balance between molecular docking accuracy and docking time cost, the Glide SP method was adopted in this study. The GlideSP scoring function is as follows:

$$\begin{array}{c} \Delta G_{\;\text{bind}\;}=C_{\;\text{lipo}‐\text{lipo}\;}\sum f\left(r_{\text{lr}}\right)+C_{\;\text{hbond}‐\text{neut}‐\text{neut}\;}\sum g\left(\Delta r\right)h\left(\Delta \alpha \right)\\ +C_{\text{hbond}‐\;\text{charged}‐\text{charged}\;}\sum g\left(\Delta r\right)h\left(\Delta \alpha \right)\\ +C_{\max‐\;\text{metal}‐\text{ion}\;}\sum f\left(r_{\text{lm}}\right)+C_{\;\text{rotb}\;}H_{\;\text{rotb}\;}\\ +C_{\;\text{polar}‐\text{phob}\;}V_{\;\text{polar}‐\text{phob}\;}+C_{\;\text{coul}\;}E_{\;\text{coul}\;}\\ +C_{\text{vdW}}E_{\text{vdW}}+\text{solvation terms } \end{array}$$

In this study, protein and small-molecule preparations were performed using the Protein Preparation Wizard and the LigPrep modules in the Glide software, respectively. Firstly, the structure of TRPV6 was optimised. This involved adding missing side chain residues and hydrogen atoms, correcting charge and overlapping atoms, and eliminating inter-atomic conflicts. Energy minimization was also performed using the OPLS3 force field at neutral pH. Docking sites were defined by referring to amino acids I575, D580, and W583 in the 6B08 structure (Bhardwaj et al., 2020). Docking grid points were generated by the Receptor Grid Generation tool in the Glide module, with the outer box side length set to 20 Å and the inner box side length set to 10 Å. The small molecule compounds in the database were prepared using the LigPreP module in the Glide software to generate three-dimensional structures and different conformations of the small molecules. All of the prepared small molecules interacted with optimized receptor proteins through ligand docking in the Glide module of the Schrodinger software. The receptor grid and ligand small molecule file path are specified in the Glide input file. The scoring function is SP. Set the maximum number of postures written from the interworking run to 5,000, and set the ‘WRITE_RES_INTERACTION’ parameter to true to generate each residue interaction item.

### 3.2 Manual screening

The top 50 compounds with the best docking scores were manually screened. The main interactions, such as steric complementarity, the protein-ligand binding mode and hydrogen bonding, were analyzed. The rationality of the docking was evaluated by observing the binding mode of the target protein structure with the small molecule ligand. The presence of hydroxyl and tertiary amine groups is usually considered beneficial, but if balmy impurity atoms are present, they are considered unfavourable. However, if a fragment containing an aromatic heteroatom also contains a carboxylic acid, the compound is considered favourable. This may be because the carboxylic acid increases the attraction of an otherwise unfavourable fragment (Kutchukian et al., 2012).

In the interaction between receptors and ligands, hydrogen bonds can be clearly identified according to the type of atom involved and the distance between them. These bonds can affect the affinity and selectivity of the two. The position of the hydrogen bond is critical for achieving efficient ligand binding, so the effect of its position on this interaction must be carefully considered in visual analysis. In addition to hydrogen bond formation, all non-interacting hydrogen bond donor and acceptor groups must be evaluated. This is because they usually result in a reduction in binding free energy due to an uncompensated free energy penalty for dissolution (Bissantz et al., 2010; Perryma et al., 2015).

AZ191 ADMET properties were predicted using the pkCSM (https://biosig.lab.uq.edu.au/pkcsm/) web servers.

### 3.3 Construction of chimera and mutants of TRPV6 ion channel

The TRPV6 mutants (I575A, D580A and W583A) and the TRPV6/TRPV1 S6 and TRPV6/TRPV2 S6 chimeras used in this experiment were constructed using Primer Premier five software to design primers (Table 3). Attention was paid to checking for primer dimers, hairpin structures and mismatches. The primers were then synthesized by Changsha Qingke Biological Company.

**TABLE 3:** Primer sequences.

Sites	Upstream primer	Downstream primers
I575A	TGC​TCA​ACC​TCC​TCG​CTG​CCA​TGA​TG	GAG​GAG​GTT​GAG​CAT​GAG​CAG​TGT​GG
D580A	CAT​GAT​GGG​CGC​CAC​TCA​CTG​GCG​AGT	CGC​CCA​TCA​TGG​CAA​TGA​GGA​GGT
W583A	CGA​CAC​TCA​CGC​GCG​AGT​GGC​CCA​T	GTG​AGT​GTC​GCC​CAT​CAT​GGC​AAT
TRPV1 S6	AGC​CAA​CTA​CAA​CGT​GGA​CCT​GCC​CAA​GGC​TGT​CTT​CAT​CAT​CCT​GTT​AC	CAA​TCT​GGG​CCC​TCC​ACA​GCT​CAT​CAA​TCT​TGT​TGA​CGG​TCT​CAC​CCA​TG
TRPV2 S6	AGC​CAA​CTA​CAA​CGT​GGA​CCT​GCC​CCG​CGG​CAT​GGT​GCT​GCT​GCT​GCT​GCT​GGC	CAA​TCT​GGG​CCC​TCC​ACA​GCT​CAT​CGG​CGA​CAC​TGT​TGA​CGG​TCT​CGC​TC
TRPV6/TRPV1 S6	CAT​GGG​TGA​GAC​CGT​CAA​CAA​GAT​TGA​TGA​GCT​GTG​GAG​GGC​CCA​GAT​TG	GTA​ACA​GGA​TGA​TGA​AGA​CAG​CCT​TGG​GCA​GGT​CCA​CGT​TGT​AGT​TGG​CT

Following primer synthesis, the target sequences were amplified *in vitro* using the PCR system. After the PCR amplification reaction, the products were fully blended with 6x L Loading Buffer and loaded onto a 1% agarose gel for electrophoretic detection. A DL12000 DNA marker was used to test the purity of the PCR products as a control. Once the target sequence has been successfully amplified, the DpnI enzyme should be used to digest the artificially added DNA template in the PCR reaction system to eliminate false positives. The extraction kit used in this study was purchased from Invitrogen as part of the PureLink™ High Pure Plasmid Filter Midiprep Kit. The TRPV channels used in this study were purchased from the Wuhan Miaolin Biological Company. The sodium and potassium channel plasmids (K_V_4.2, K_V_4.3, Na_V_1.5, HERG, etc.) were obtained from our laboratory. Calculate the inhibition rate of the compound on ion channel activity at different concentrations using the following equation: Y=Bottom+ (Top−Bottom)/​ (1 + 10 (logIC_50_​−[C]) × HillSlope). Use the GraphPad Prism 8 software to determine the IC_50_ value.

### 3.4 Cell culture

The HEK293T cells were purchased from the Cell Bank at the Shanghai Institute of Life Sciences, Chinese Academy of Sciences. The other cells were purchased from Wuhan Punosai Life Technology Co., Ltd. Complete medium formulation: Add 50 mL of fetal bovine serum (FBS, Gibco) and 5 mL of double antibody (PS, Gibco) to 450 mL of medium (Gibco), then store the mixture at 4 °C. The cells were maintained under conditions of 21% oxygen, 5% carbon dioxide and 37 °C (Table 4).

**TABLE 4:** Cell culture conditions.

Name of cell	Culture conditions
RT4	MCCOY’s 5A + 10%FBS + 1%PS
HT-1376	DMEM + 10% FBS + 1%PS
HT29	DMEM + 10%FBS + 1%PS
HCT116	DMEM + 10%FBS + 1%PS
K562	DMEM + 10%FBS + 1%PS
Hela	RPMI 1640 + 10%FBS + 1%PS
MBA-MD231	DMEM + 10%FBS + 1%PS
SKOV3	RPMI 1640 + 10%FBS + 1%PS
LNCaP	RPMI 1640 + 10%FBS + 1%PS
22RV1	DMEM + 10%FBS + 1%PS
HEK-293T	DMEM + 10%FBS + 1%PS

### 3.5 Whole-cell recording clamp experiments

The experimental electrodes were made from microelectrode glass capillary tubes using a PC-10 Electrode Puller and a two-step temperature pulling process. The resistance value was controlled at 2.5–3.5 MΩ. The EPC-10 USB amplifier was used to collect the data, and the PatchMaster software was used to control and record it. The cell culture fluid was discarded, after which 1.5 mL of extracellular fluid was added to the dish (Table 5). The dish was then placed under an inverted microscope and into the reference electrode. Intracellular fluid was added to the electrode. Air bubbles were gently popped by hand and positive pressure was applied after the electrode was fixed in place. Under the microscope, a single cell with green fluorescence, a smooth surface and normal morphology was selected and placed in the centre of the field of view. Slowly move the glass electrode to make contact with the extracellular fluid to compensate for the liquid-joint potential, ensuring that the inlet resistance is between 2.5 and 3.5 MΩ. Use the micromanipulator to slowly move the electrode to the centre of the cell and gently press the middle of the cell with the electrode. The cell will undergo a tiny deformation. Open the three-way valve and gently suck on the hose to create a slight negative pressure. This will cause the electrode and the cell membrane to come into close contact, resulting in a gradual increase in resistance to the GΩ level. Switch to WHOLE-CELL mode and compensate for fast capacitance. Open the three-way valve to break the membrane. A sharp slow capacitance peak will then appear on the oscilloscope interface. Compensate for the series resistance and leakage current. Once the resistance value has stabilized, apply the stimulus according to the selected mode and record the channel current.

**TABLE 5:** Formula of intracellular and extracellular fluid.

Name	Ingredients
Na^+^ (extracellular)	NaCl 140 mM, KCl 5 mM, CaCl_2_ 2 mM, MgCl_2_ 1 mM, HEPES 10 mM, Glucose 10 mM, pH = 7.4
Na^+^ (intracellular)	CsF 140 mM, NaCl 10 mM, HEPES 10 mM, EGTA 1 mM, pH = 7.4
K^+^ (extracellular)	NaCl 140 mM, KCl 5 mM, CaCl_2_ 2 mM, MgCl_2_ 1 mM, HEPES 10 mM, Glucose 10 mM, pH = 7.4
K^+^ (intracellular)	EGTA 11 mM, KCl 140 mM, MgCl_2_ 2.5 mM,HEPES 10 mM, pH = 7.4
TRPV1-TRPV3 channel	NaCl 130 mM,HEPES 3 mM, EGTA 0.2 mM, pH = 7.2
TRPV6 (extracellular)	NaCl 140 mM, CsCl 6 mM, MgCl_2_ 1 mM, HEPES 10 mM, Glucose 10 mM, pH = 7.4
TRPV6 (intracellular)	Cs-Asp 100 mM, CsF 20 mM, EGTA 10 mM, MgCl_2_ 3 mM, Na-ATP 3 mM, HEPES 20 mM, pH = 7.4

### 3.6 CCK-8 assay

These cancer cell lines were seeded at a density of 3,000 per well in 96-well plates, with a total volume of 100 μL, respectively. Following overnight adherence to the wall, the compound AZ191 was diluted using a complete medium gradient and added to the wells. A control group without drug treatment was also set up. The positive control drug selected was paclitaxel, a common broad-spectrum anti-tumor agent. Subsequently, the plates were placed in the chamber (37 °C, 1% O_2_, 5% CO_2_) for the duration of the experiment. After 48 h, the original medium was aspirated, and 100 µL of basal medium containing 10% CCK-8 (TargetMol, China) was added to each well. The plate was then incubated for a further 2 h. The optical density (OD) value was then quantified using an enzyme-labelling instrument (Bio-Tek Cytation5) at a wavelength of 450 nm. Repeat the experiment three times and calculate the average. The cell survival rate is calculated as: (Absorbance of treated group/Absorbance of control group) × 100%. Use the GraphPad Prism 8 software to determine the IC_50_ value.

### 3.7 Statistics

All data were expressed as mean ± standard deviation (SD), and statistical analysis was performed using GraphPad Prism 8 software. All experiments were conducted at least three times. The data were subjected to one-way analysis of variance (ANOVA) to ascertain the disparities between the two groups. For all statistical tests, a p-value of less than 0.05 was considered statistically significant.

## 4 Conclusion

Currently, tumors are still the leading disease endangering human life and health. The utilization of small molecule targeted agents continues to represent the primary foundation of cancer treatment. An increasing body of evidence from a range of studies suggests that TRPV6 has a pivotal function in the development of tumors, acting as a calcium-selective channel. Consequently, TRPV6 inhibitors represent a highly promising research avenue for the development of novel anti-tumor drugs. Nonetheless, the development of TRPV6 inhibitors remains in the preliminary stage. This study employed a combination of structure-based virtual screening and systematic biochemical experiments, which proved to be a successful methodology for the identification of a novel small-molecule inhibitor of TRPV6, designated as AZ191. Additionally, the experimental results confirmed that the aspartate residue at position 580 of the S6 fragment of the TRPV6 structure is essential for AZ191 to inhibit TRPV6. It is noteworthy that AZ191 displays a wide-ranging anti-tumor effect by impeding TRPV6. In conclusion, this study further confirms that TRPV6 is a promising drug target for tumor treatment and provides a novel lead compound for the subsequent development of TRPV6 inhibitors.

## Data Availability

The original contributions presented in the study are included in the article/supplementary material, further inquiries can be directed to the corresponding authors.
